# Supplementary material for: Hippocampal GABA enables inhibitory control over unwanted thoughts
Source: Nat Commun. 2017 Nov 3;8:1311. doi: 10.1038/s41467-017-00956-z (PMC5670182; doi:10.1038/s41467-017-00956-z)
Supplement: Supplementary file 1 — Supplementary Information [file 41467_2017_956_MOESM1_ESM.pdf]

Supplemental Information for:

Hippocampal GABA enables inhibitory control over unwanted thoughts

Taylor W. Schmitz*, Marta M. Correia, Catarina S. Ferreira, Andrew P. Prescot, & Michael C. Anderson*

correspondence to: T.W.S. (tws35@cam.ac.uk) or M.C.A. (Michael.Anderson@mrc-cbu.cam.ac.uk)

Table of Contents

I. Supplemental Figure S1

II. Supplemental Figure S2

III. Supplemental Table S1

IV. Supplemental Methods

1. Behavioral analysis
2. MRI analysis
    a. Region of interest (ROI) specification
    b. Psychophysiological interaction (PPI) analysis
    c. Dynamic causal modeling (DCM) analysis
3. ^{1}H MRS analysis
    a. Spectral fitting
    b. ROI tissue segmentation
    c. Water suppression

V. Supplemental Notes

1. Weighted least squares regression analyses (WLSR)
    a. WLSR modeling hippocampal line width
    b. WLSR modeling hippocampal GABA fitting error (Cramér-Rao lower bound)
    c. WLSR modeling inter-scan interval
2. Dynamic Causal Modeling
    a. Whole group analysis

Figure S1.

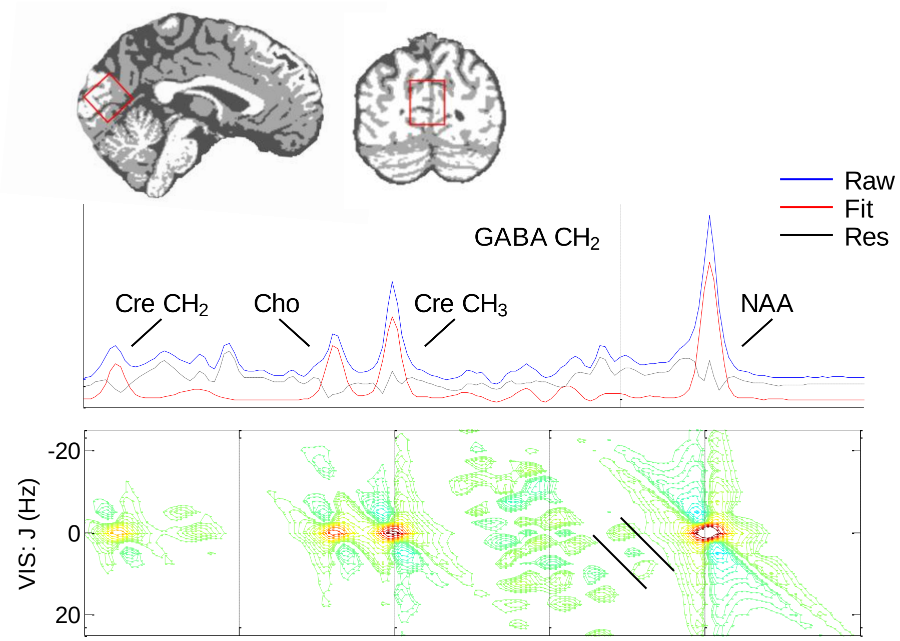

^{1}H MRS quantification of GABA concentrations in visual cortex. (Top). Positions of the visual cortical voxel are displayed on sagittal (top row) and coronal (bottom row) slices extracted from an example subject's tissue-segmented structural scan. (Middle) An example of the ^{1}H MRS spectra displayed in one dimension. Blue line: raw metabolite spectra for an example subject. Red line: ProFit basis functions for singlet (one-peak) metabolites, including Creatine (Cre), Choline (Cho), and N-acetyl aspartate (NAA). Black line: residuals after fitting. Note the GABA CH_2 methylene group at 2.28 PPM is invisible on the 1D plot. (Bottom) Plotted for the visual cortical voxel (*N*=20) are the fitted spectra (averaged over all subjects) of the same four metabolites, but now spread along two dimensions, the J-resolved axis (±20 Hz) plotted and the chemical shift axis (1.5—4 parts per million; ppm). Both plots use identical scaling. Colors indicate minimum (blue) and maximum (red) height of spectral contours (arbitrary units). The GABA CH_2 methylene group is visible at 2.28 ppm (diagonal lines).

Figure S2.

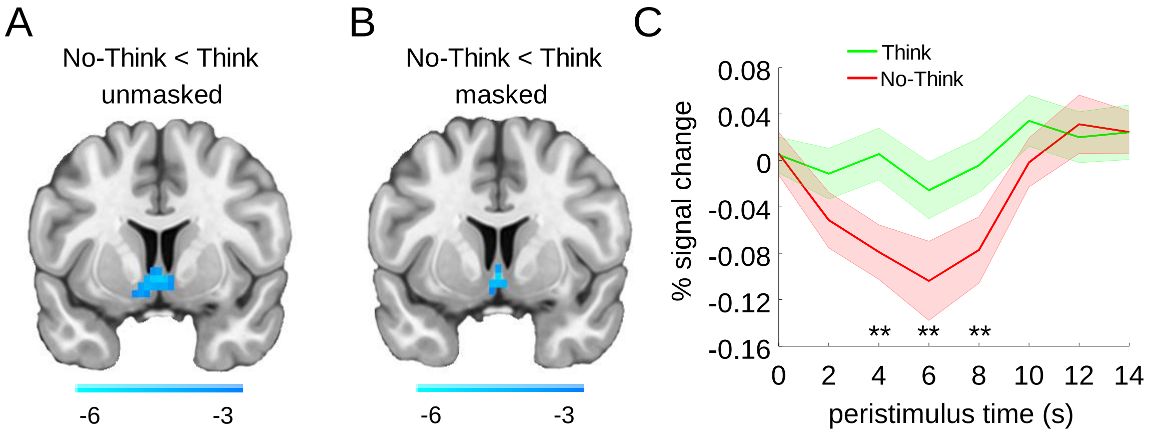

Modulation of the medial septal nucleus by suppression. (A) Group whole-brain contrast for No-Think<Think. Thought suppression down-regulated septal nucleus activity. Septal activations are illustrated on a coronal slice in MNI space. Activations are derived from an uncorrected cluster-defining threshold ($p<0.001$), with cluster level false discovery rate $p<0.05$. Color bars demarcate T-statistics. (B) Group masked activations for the No-Think<Think contrast, with activity constrained to the septal nucleus ROI volume (small volume FWE corrected $p<0.05$). (C). A priori region of interest analysis: Group hemodynamic time-courses were attenuated in septal nucleus by thought suppression (No-Think) relative to retrieval (Think). **$p<0.01$.

Table S1.

Low and High Hippocampal GABA subgroups

	Subgroups		
**Demographics**	**low GABA**	**high GABA**	***t*-test**
N (Male, Female)	9 (2, 7)	9 (3, 6)	*t=0.50, p=0.62*
Age in years (SD)	24.55 (5.34)	25.55 (4.22)	*t=0.44, p=0.67*
**Cognitive measure**			
Criterion Memory Test (%)	74.8±5.65	70±5.11	*t=0.63, p=0.54*
SSRT (ms)	318.88±18.02	323.64±13.42	*t=0.21, p=0.84*
Go RT (ms)	581.17±16.70	572.23±12.17	*t=0.43, p=0.67*
Go Accuracy (%)	94.16±1.25	94.23±0.76	*t=0.05, p=0.96*
**^{1}H MRS measure**			
HIP GABA/Cre	0.14±0.01	0.24±0.01	*t=6.08, p<0.001*
DLPFC GABA/Cre	0.18±0.02	0.16±0.01	*t=1.25, p=0.23*
VIS GABA/Cre	0.17±0.01	0.16±0.01	*t=0.62, p=0.55*

SSRT = Stop-signal response time, RT = response time to Go trials. Tabled values are the mean of each subgroup ± standard error of the mean. All t-statistics are independent samples t-tests, with 8 degrees of freedom, on a 2-tailed alpha.

Supplemental Methods

Behavioral analysis:

The stop-signal reaction time (SSRT) was estimated according to the blocked integration method provided by the independent-race model[1,2]. In this model, SST performance is modeled as a race between a go process (triggered by the presentation of the go stimulus) and a stop process (triggered by the presentation of a stop signal). The stop signal occurs after a variable interval, the stop-signal delay (SSD). The point at which the stop process finishes is estimated by integrating the response time (RT) distribution and finding the point at which the integral equals the probability of responding, p(respond|signal), for a specific SSD. SSRT is then calculated by subtracting SSD from the finishing time. We estimated SSRT for each of the 8 sessions separately before averaging together as a whole, i.e. blocked integration[3], to minimize underestimation of the SSRT due to gradual slowing of RTs over the course of the experiment.

Final recall for No-Think, Think, and Baseline items was conditionalized relative to the number of successfully learned words, as in prior work[4]. Thus, the conditionalized analyses indicate the percentage of words remembered conditional on correct initial learning. To examine relationships between forgetting and brain activation, we expressed below-baseline forgetting as recall performance for Baseline minus performance for No-Think items. This was done separately for the same probe (SP) and independent probe (IP) test data[5]. We then averaged the forgetting scores of the two tests to get our index of suppression-induced forgetting (SIF).

MRI analysis

Regions of Interest (ROI) Specification

Two distinct *a priori* regions of interest (ROI) were defined for the multimodal imaging analyses: the right dorsolateral prefrontal cortex (DLPFC) and the right hippocampus (HIP). For the DLPFC ROI, we first derived a binarized map of the [No-Think >Think] contrast in the direct

suppression group from Benoit and Anderson[6]. We then isolated from this map the DLPFC cluster, which centered on the following MNI coordinates: x=36, y=38, z=31. For each participant, the DLPFC ROI was transformed into native space using the deformation field produced at the non-linear warping step of their fMRI data pre-processing (see below). For the HIP ROI, we manually traced the hippocampus in native space for each participant, using established anatomical guidelines[7,8], and the itk-SNAP segmentation toolbox[9]. We further parcellated the HIP ROI into anterior and posterior segments, at the first coronal slice in which the uncal apex was visible[10] in order to explore possible variation in BOLD responses across the longitudinal axis[11-13].

The anatomical landmarks for the DLPFC and HIP ROIs were used to guide positioning of the MRS voxels. For the DLPFC MRS voxel (25x25x25 mm^3), we used the landmarks surrounding the BA46/BA9 DLPFC cluster from Benoit and Anderson[6]. In the axial plane, the posterior face of the ROI was positioned ~1 cm anterior to the precentral sulcus. In the coronal place, the ROI was then rotated obliquely until it aligned with the lateral surface of the middle frontal gyrus and the superior frontal sulcus. For the HIP MRS voxel (10x10x40 mm^3), we used a subset of the same anatomical guidelines that were used for manually tracing the hippocampus to identify the anterior, lateral, and inferior edges[7,8]. To improve visualization of the hippocampus, the structural T1-weighted volume was first resliced coronally in plane with the longitudinal hippocampal axis. In the sagittal plane, the anterior face of the ROI was positioned posterior to the white matter border of the alveus, and rotated obliquely until it aligned with the longitudinal hippocampal axis. In the coronal plane, the ROI was then centered on the triangular grey area of the pes hippocampi.

For the multimodal control analysis, we defined an a priori region of interest (ROI) in visual cortex (25x25x25 mm^3) using the same landmarks described in Edden et al.[14]. In the axial plane, the VIS voxel was centered on the midline of the visual cortices. In the sagittal plane, the

ROI was then rotated until it aligned with the cerebellar tentorium and parieto-occipital sulcus (see Figure S1).

For the a priori fMRI analysis of the basal forebrain, we used a stereotaxic probabilistic anatomical map of the basal forebrain nuclei, derived from the SPM Anatomy Toolbox[15,16]. Given our interest in the septohippocampal pathway, we restricted our analysis to the septal nucleus of the basal forebrain (see Figure S2).

Psychophysiological interaction (PPI) analysis:

We conducted a PPI analysis[17] to test the hypothesized relationship between right HIP coupling and retrieval suppression. In each subject, the anatomically defined hippocampal ROI was binarized into a mask image. To ensure that extracted signal for the PPI reflected meaningful voxels, i.e. those differentiating No-Think and Think conditions, we computed the top 60% of voxels within the masking region that expressed a main effect (Think versus No-Think F-contrast) using custom Matlab scripts (version R2010b, The MathWorks, Natick, MA). Uninformative voxels were discarded from the masking region. The 'thresholded' hippocampal mask was then converted to a volume of interest (VOI), from which we extracted the first eigenvariate, adjusted for main effects of task. The PPI regressor was then calculated as the volume-by-volume product of the deconvolved VOI time series and a binary vector coding for No-Think and Think trials[18]. The three PPI regressors were then separately reconvolved with the canonical HRF and entered into a first-level GLM, with all other design specifications identical to the original GLM (e.g. separately modeled SST and Error regressors). Contrasts were specified over the PPI regressor for random effects analysis (1 and −1), reflecting activations either positively or negatively related to the PPI interaction term, respectively.

Dynamic Causal Modeling (DCM) analysis:

To test the putative fronto-hippocampal pathway supporting retrieval suppression, we modeled the effective connectivity between DLPFC and HIP ROIs using DCM12. DCM explains regional effects in terms of dynamically changing patterns of connectivity among nodes of a pre-specified network during experimentally induced contextual changes[19]. Note that modeling interactions between nodes does not presuppose that these regions exhibit monosynaptic connections. Rather, the resulting coupling parameters represent their effective connectivity, which may well be mediated by relay nodes[19-21]. For each subject, we extracted signal from the DLPFC node according to the same protocol used for the HIP VOI in the PPI analysis. The HIP signal modeled in the PPI and DCM analyses was thus identical. For bilinear DCM analysis, three parameters are specified for each model: (1) Intrinsic connections (based on hypothesized mono- and poly-synaptic connections between the nodes), (2) bilinear modulation of connections by experimental conditions, and (3) driving inputs into nodes from experimental conditions. Model fitting was achieved by adjusting the model parameters to maximize the free-energy estimate of the model evidence[19]. Neural activity from each node was extracted and Bayesian model selection (BMS) was then used to identify the family that could account best for the data[22]. A random-effects approach was taken, since it does not assume that the optimal model will be the best for each individual[21]. This analysis reports the exceedance probability, i.e., the probability to which a given model is more likely than any other included model to have generated the data from a randomly selected participant.

For the subgroup analysis comparing model parameters, we performed Bayesian model averaging over the bidirectional family model space to obtain, for each subgroup, a single set of estimates for model parameters ii (modulatory connections) and iii (driving inputs).

^{1}H MRS analysis

Spectral Fitting

The basis set provided by ProFit[23] comprised nineteen metabolites (see supplemental methods) including creatine (Cre), GABA, and glutamate. N-acetyl aspartate (NAA), glycerophosphorylcholine (GPC), phosphorylcholine (PCh), alanine (Ala), aspartate (Asp), GABA, glucose (Glc), Gln, Glutamate (Glu), Gly, GSH, Lac, myoinositol (Ins), N-acetyl aspartylglutamate (NAAG), phosphoethanolamine (PE), taurine (Tau), scyllo-inositol (sI), and ascorbic acid (Asc). The Cre methylene (CH_2) and methyl (CH_3) protons were fitted separately whereas the separate GPC and PCh peaks ultimately were considered as a composite resonance.

ROI tissue segmentation

Skull stripping and whole brain tissue-type segmentation of the MP-RAGE volumes was performed using BET[24] and FAST[25], respectively, which are tools provided with the freely-available FMRIB software library (FSL)[26]. Matlab (version R2010b, The MathWorks, Natick, MA) functions written by one of the authors (A.P.P.) were used to extract the 3D volume corresponding to the positioned MRS voxel to obtain within-voxel gray matter (GM), white matter (WM) and cerebrospinal fluid (CSF) tissue content for each subject.

Water suppression

A three-pulse water elimination through T1-effects[27] scheme was interleaved with the outer-volume suppression (OVS) module for global water suppression. In addition, water unsuppressed 2D ^{1}H MRS data were acquired from each voxel with a single acquisition recorded for each TE step (acquisition time 3 min 28 sec). The RF transmitter carrier frequency was set to 3.0 and 4.7 ppm for water suppressed and unsuppressed data, respectively. Before channel recombination, eddy current distortions initially were accounted for using a previously

reported method[28]. The residual water signal was removed from each row of water suppressed 2D matrices using a Hankel singular value decomposition (HSVD) routine[29] written in MATLAB. Finally, the 2D matrix was reformatted to produce the individual file types required for ProFit read-in.

Supplemental Notes

1. Weighted least squares regression analyses (WLSR)

We assessed the impact of two limitations inherent to our ^{1}H MRS data, which concern the relatively lower field homogeneity of the hippocampus voxel (compared to DLPFC and visual cortical voxels), and also the variable interval between fMRI and ^{1}H MRS acquisitions, which ranged from 1—111 days across participants. To do so, we re-analyzed our primary findings with WLSR, which gives each data point an amount of influence over the parameter estimates proportionate to its 'quality.' Below we describe how quality was parameterized for each model. In general, the WLSR analyses demonstrate that the relationships did not change substantially when carefully adjusting the amount of influence of each datapoint over the parameter estimates according to the line widths of the hippocampal GABA signal, or inter-scan interval.

WLSR modeling hippocampal line width

Under the assumption that broader line widths (Hz) obtained from the shims of the hippocampal voxel reflect lower quality data, we used weighted least squares regression to give each data point its proper amount of influence over the parameter estimates. Each subject was therefore precisely weighted by subtracting their hippocampal line width from a constant value, ensuring all weights were positive values. Individuals with higher values (broader line widths) contributed proportionally smaller weights to the model. Below we show the standardized coefficients (Betas) for the primary relationships reported in Table 1, using symmetrical linear regression models that differed only by inclusion of the weighting variable ('weighted' versus 'unweighted').

	Unweighted	Weighted
**HIP GABA / HIP BOLD**	Beta	Beta
NT	-0.46	-0.43
T	-0.60	-0.59
**HIP GABA / Behavior**		
SIF	0.59	0.62
**HIP GABA / PPI**		
DLPFC	-0.61	-0.59

Of the observed significant relationships with hippocampal GABA, statistical inference on only one relationship was affected by weighting with line width (the Hip GABA/ Hip BOLD relationship during NT), through the actual magnitude of this change was quite small.

WLSR modeling hippocampal GABA fitting error (Cramér-Rao lower bound)

The Cramér-Rao lower bound (CRLB) values reflect the threshold of uncertainty for the error associated with model fitting. For the hippocampal GABA fits, we assumed higher CRLB reflected lower quality data (mean=22.86, SD=9.6). Each subject was therefore precisely weighted by subtracting their hippocampal GABA CRLB value from a constant value, ensuring all weights were positive values. Individuals with higher CRLB values therefore contributed proportionally smaller weights to the model. Below we show the standardized coefficients (Betas) for the primary relationships reported in Table 1, using symmetrical linear regression models that differed only by inclusion of the weighting variable ('weighted' versus 'unweighted').

	Unweighted	Weighted
**HIP GABA / HIP BOLD**	Beta	Beta
NT	-0.46	-0.41
T	-0.60	-0.54
**HIP GABA / Behavior**		
SIF	0.59	0.59
**HIP GABA / PPI**		
DLPFC	-0.61	-0.61

Of the observed significant relationships with hippocampal GABA, inference on only one relationship was affected by weighting with CRLB (The Hip GABA/Hip BOLD relationship during NT). Again, the actual magnitude of this change was quite small.

WLSR modeling inter-scan interval

We next assessed whether the relationships reported in this manuscript were affected when subjects were weighted according to their inter-scan interval, under the assumption that longer intervals reflect lower quality data. Each subject was precisely weighted by the number of days between the fMRI and MRS acquisition, by subtracting this interval from a constant to ensure positive values. Individuals with longer intervals therefore contributed proportionally smaller weights to the model. Below we show the standardized coefficients (Betas) for the primary relationships reported in Table 1, using symmetrical linear regression models that differed only by inclusion of the weighting variable (‘weighted’ versus ‘unweighted’).

	**Unweighted**	**Weighted**
**HIP GABA / HIP BOLD**	Beta	Beta
NT	-0.46	-0.50
T	-0.60	-0.63
**HIP GABA / Behavior**		
SIF	0.59	0.63
**HIP GABA / PPI**		
DLPFC	-0.61	-0.55

Of the observed significant relationships with hippocampal GABA, none were affected by weighting with Interval; in fact most were slightly improved.

2. Dynamic Causal Modeling

Whole group analysis

According to our hypothesis, only memory operations and, specifically, retrieval suppression, should drive DLPFC-hippocampal network dynamics. We tested these predictions using hierarchical random effects Bayesian model selection to compare model families grouped according to a shared feature of interest [22]. In the first step, we compared families grouped by modulatory connection: bottom-up, top-down, bidirectional, or none. Consistent with prior work[6,30], the bidirectional family obtained the most evidence. We found a 91% likelihood that the bidirectional model generated participants' data (known as the exceedance probability, or EP). For any given participant, we found a 61% likelihood of identifying the bidirectional model as the generator of the data (known as the posterior probability, or PP). Exceedance and posterior probabilities for the remaining families were, respectively, as follows: No-modulation (1% and 9%), bottom-up (7% and 20%), top-down (1% and 10%). By contrast, a parallel analysis, substituting the No-Think and Think conditions with Stop and Go conditions, failed to provide evidence favoring any modulatory family over the null family. Exceedance and posterior probabilities for the four modulatory families were, respectively, as follows: No-modulation (28% and 27%), bottom-up (35% and 31%), top-down (24% and 24%), bidirectional (13% and 17%).

References

1. Logan, G.D. & Cowan, W.B. On the ability to inhibit thought and action: A theory of an act of control. *Psychological review* **91,** 295–327 (1984).

2. Verbruggen, F. & Logan, G.D. Models of response inhibition in the stop-signal and stop-change paradigms. *Neurosci Biobehav Rev* **33,** 647-661 (2009).

3. Verbruggen, F., Chambers, C.D. & Logan, G.D. Fictitious inhibitory differences: how skewness and slowing distort the estimation of stopping latencies. *Psychol Sci* **24,** 352-362 (2013).

4. Anderson, M.C. *et al.* Neural systems underlying the suppression of unwanted memories. *Science* **303,** 232-235 (2004).

5. Anderson, M.C. & Green, C. Suppressing unwanted memories by executive control. *Nature* **410,** 366-369 (2001).

6. Benoit, R.G. & Anderson, M.C. Opposing mechanisms support the voluntary forgetting of unwanted memories. *Neuron* **76,** 450-460 (2012).

7. Duvernoy, H.M., *The human hippocampus: Functional anatomy, vascularization and serial sections with MRI*, Third ed. (Springer, New York, 2005).

8. Pruessner, J.C. *et al.* Volumetry of hippocampus and amygdala with high-resolution MRI and three-dimensional analysis software: minimizing the discrepancies between laboratories. *Cereb Cortex* **10,** 433-442 (2000).

9. Yushkevich, P.A. *et al.* User-guided 3D active contour segmentation of anatomical structures: significantly improved efficiency and reliability. *Neuroimage* **31,** 1116-1128 (2006).

10. Weiss, A.P., Dewitt, I., Goff, D., Ditman, T. & Heckers, S. Anterior and posterior hippocampal volumes in schizophrenia. *Schizophr Res* **73,** 103-112 (2005).

11. Chase, H.W. *et al.* Evidence for an anterior-posterior differentiation in the human hippocampal formation revealed by meta-analytic parcellation of fMRI coordinate maps: focus on the subiculum. *Neuroimage* **113,** 44-60 (2015).

12. Qin, S. *et al.* Large-scale intrinsic functional network organization along the long axis of the human medial temporal lobe. *Brain Struct Funct* (2015).

13. Robinson, J.L. *et al.* Neurofunctional topography of the human hippocampus. *Human brain mapping* **36,** 5018-5037 (2015).

14. Edden, R.A., Muthukumaraswamy, S.D., Freeman, T.C. & Singh, K.D. Orientation discrimination performance is predicted by GABA concentration and gamma oscillation frequency in human primary visual cortex. *J Neurosci* **29,** 15721-15726 (2009).

15. Eickhoff, S.B. *et al.* A new SPM toolbox for combining probabilistic cytoarchitectonic maps and functional imaging data. *Neuroimage* **25,** 1325-1335 (2005).

16. Zaborszky, L. *et al.* Stereotaxic probabilistic maps of the magnocellular cell groups in human basal forebrain. *Neuroimage* **42,** 1127-1141 (2008).

17. Friston, K.J. *et al.* Psychophysiological and modulatory interactions in neuroimaging. *Neuroimage* **6,** 218-229 (1997).

18. Gitelman, D.R., Penny, W.D., Ashburner, J. & Friston, K.J. Modeling regional and psychophysiologic interactions in fMRI: the importance of hemodynamic deconvolution. *Neuroimage* **19,** 200-207 (2003).

19. Friston, K.J., Harrison, L. & Penny, W. Dynamic causal modelling. *Neuroimage* **19,** 1273-1302 (2003).

20. Stephan, K.E., Friston, K.J. & Frith, C.D. Dysconnection in schizophrenia: from abnormal synaptic plasticity to failures of self-monitoring. *Schizophr Bull* **35,** 509-527 (2009).

21. Stephan, K.E. *et al.* Ten simple rules for dynamic causal modeling. *Neuroimage* **49,** 3099-3109 (2010).

22. Penny, W.D. *et al.* Comparing families of dynamic causal models. *PLoS Comput Biol* **6,** e1000709 (2010).

23. Schulte, R.F. & Boesiger, P. ProFit: two-dimensional prior-knowledge fitting of J-resolved spectra. *NMR Biomed* **19,** 255-263 (2006).

24. Smith, S.M. Fast robust automated brain extraction. *Human brain mapping* **17,** 143-155 (2002).

25. Zhang, Y., Brady, M. & Smith, S. Segmentation of brain MR images through a hidden Markov random field model and the expectation-maximization algorithm. *IEEE transactions on medical imaging* **20,** 45-57 (2001).

26. Smith, S.M. *et al.* Advances in functional and structural MR image analysis and implementation as FSL. *Neuroimage* **23 Suppl 1,** S208-219 (2004).

27. Ogg, R.J., Kingsley, P.B. & Taylor, J.S. WET, a T1- and B1-insensitive water-suppression method for in vivo localized 1H NMR spectroscopy. *Journal of magnetic resonance. Series B* **104,** 1-10 (1994).

28. Klose, U. In vivo proton spectroscopy in presence of eddy currents. *Magn Reson Med* **14,** 26-30 (1990).

29. Cabanes, E., Confort-Gouny, S., Le Fur, Y., Simond, G. & Cozzone, P.J. Optimization of residual water signal removal by HLSVD on simulated short echo time proton MR spectra of the human brain. *Journal of magnetic resonance* **150,** 116-125 (2001).

30. Benoit, R.G., Davies, D.J. & Anderson, M.C. Reducing future fears by suppressing the brain mechanisms underlying episodic simulation. *Proc Natl Acad Sci U S A* **113,** E8492-E8501 (2016).
